# Regulatory, safety and economic considerations of over-the-counter medicines in the Indian population

**DOI:** 10.1007/s44250-023-00032-y

**Published:** 2023-05-22

**Authors:** Prashant Narang, Vandana Garg, Atul Sharma

**Affiliations:** 1 Medical Affairs, Haleon (Formerly GSK Consumer Healthcare Pte Ltd.), 10th Floor, One Horizon Center, Golf Course Road, DLF Phase 5, Gurugram, Haryana 122002 India; 2 Medical Affairs, Haleon (Formerly GSK Consumer Healthcare Pte Ltd.), Singapore, Singapore

**Keywords:** Nonprescription drugs, OTC drug policy, Rx to OTC, Self-medication

## Abstract

**Background and purpose of review:**

Over-the-counter (OTC) medication has been an integral component of an established health care system but their easy accessibility might pose significant risks. This review has attempted to highlight the present scenario of OTC utilization in India, regarding standard practices followed globally. An attempt has also been made to highlight the lifecycle of a prescription and OTC medicine and the benefits and regulatory process involved in the prescription-to-OTC switch.

**Findings:**

A paradigm shift has been observed in self-medication practice with OTC medicines in recent times and has become a widespread practice worldwide. Numerous key drivers, such as increasing consumer awareness, broader consumer access to essential medication, and socio-economic benefits to the public health care system, have advocated this practice. On the other hand, self-medication using OTC is also inextricably linked with inevitable risks such as excessive drug dosage, polypharmacy, drug abuse, and drug interactions. Nevertheless, these issues could be further regulated by employing a defined OTC framework. The government of India has recognized the utmost need to develop a robust policy framework for the effective utilization of OTC drugs. Also, various initiatives toward modifying existing laws or developing new OTC drug policies has been taken.

**Conclusion:**

Prioritizing the utmost safety of the consumers and evident need of strong regulatory framework with respect to OTC drugs, the term “OTC” has been recommended as a distinct category of drugs by Government of India. This review has highlighted various factors that can play an essential role in OTC utilization and can be considered during policy reformation.

## Introduction

Self-medication practice with non-prescription drugs [sometimes referred to as over-the-counter (OTC) medicines] is increasing worldwide, and the prevalence rate has been assessed to be in-between 11.2 and 93.7%, depending on the target population and country [1, 2]. In India, the range was between 8.3 and 92% among the lower and middle-income groups. The major driving force behind raising self-medication practices are raising tendency to self-manage symptoms, cost escalation in the health care system, and easy accessibility of health-related information on the internet and social media advertisements and communications of OTC drugs. Moreover, the ease of visiting a pharmacy in preference to a hospital visit is also considered the most common reason for self-medication [3].

With an increase in self-care and self-medication practice, the trend towards deregulation of more medicines with well-established safety and efficacy profiles to OTC status is also increasing, and this increase has the support of policymakers, Healthcare Professionals (HCPs), consumers, and pharmaceutical industries. Self-medication utilizing OTC medications not only provides patients with a higher degree of self-governance in managing minor illnesses but also benefits in economizing the health care cost. The availability of OTC for self-medication in the U.S. healthcare system saved $102 billion annually, including $25 billion in drug cost savings and $77 billion from clinical visits [4]. This accelerating trend and economic benefits are posing a considerable impact worldwide, specifically in developing countries like India. An Indian study investigating the 'Value of OTC in India' reported approximately Rs.30,730 cr annual savings and improvement in overall health outcomes with OTC availability [5]. A few recent articles and media reports highlighted that the Indian government is now giving utmost consideration to OTC regulations, and the term "OTC" has been recommended as a distinct category of drugs [6].

In India, the drugs that do not come under the prescription medicines category are generally considered over-the-counter medicines. Thus, a well-regulated category of OTC medicines, patient awareness programs, and pharmacists and pharmaceutical companies' participation are imperative to optimize the use of OTC medicines in India. This review article attempts to throw light on the trends of OTC medicines utilization, benefits and concerns, and approaches, including evolving regulatory landscapes, to address these concerns in India against the globally prevalent practices. In addition, the review presents a comprehensive view of various determinant factors such as the impact of Rx to OTC switch, global OTC market, consumer awareness, and impact on the special population that need to be considered during policy reformation.

### Defining OTC medications

Over-the-counter (OTC) medicines are therapeutic products that can be sold directly to consumers without a prescription in compliance with the regulations posed by each country. OTC medications are mainly used as a first-line/initial therapy approach covering many minor and self-limiting conditions, including but not limited to the common cold, headaches, musculoskeletal pain, heartburn, and allergies. The selection of medicine in the OTC category is mainly based on the safety and efficacy of any medicine. According to World Health Organization (WHO), self-medication has been considered an integral part of the evolving healthcare system, focusing primarily on consumers’ awareness, education, and socio-economic status [7].

## Lifecycle of prescription and OTC medicines

The drugs are classified into prescription or non-prescription drug categories based on their inherent toxicity, intended use, dosage form, posology, and safety. The drug's safety information obtained from market experiences plays a significant role in reclassifying the drug in a downward or upward status. Drugs for catastrophic diseases are mainly categorized in the prescription drug category, thus, consistently regulated throughout their lifecycle in contrast to the non-prescription drug category (OTC).

A new drug application (NDA) may be submitted for a direct-to-OTC drug product. However, many FDA-approved OTC drug products (i.e., OTC products that have an approved NDA) begin their lifecycle as NDA-approved prescription drugs and eventually switch to OTC status under the NDA provisions. This process is commonly referred to as an Rx-to-OTC switch. However, switching a new chemical entity or a prescription product, which meets the essential criteria of inherent toxicity, intended use, dosage form, posology, and safety, requires the following additional criteria to advocate the change of status to non-prescription sale [8].

**Fig. 1:**
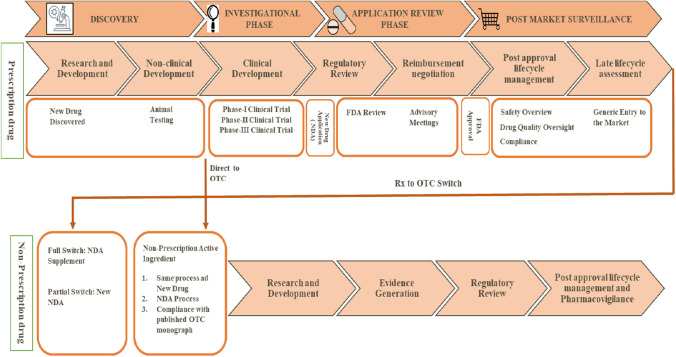
Comparison of the life cycle of prescription medicine and non-prescription drug

1. Extensive or high-volume use of product.
2. The product has been prescription marketed for at least five continuous years in the same country in sufficient quantity. However, the appropriate time considered on prescription for a product may varies for example, up to 10 years in the Philippines, 6 years in Japan, 3 years in New Zealand and no time specified in the European Union. In India, a new drug shall continue to be considered as new drug for a period of 4 years from the date of its first approval [9]. The basic notion of 5 years of prescription marketing is based upon the fact that with the help of effective safety monitoring system, the adverse events associated with products have been recognized in the first 5 years.
3. Its adverse events give no cause for concern, and their frequency has not increased unduly during the marketing period. Figure 1 compare the life cycle of prescription medicine and non-prescription drug.

Rx-to-OTC switch provides several benefits to consumers, patients, regulators, and pharmaceutical companies. Some of these benefits provided by Rx to OTC switch are discussed in Table 1 [10–18].

**Table 1:** Benefits associated with Rx-to-OTC switch to consumers and patients, regulators and pharmaceutical companies

Beneficiaries	Benefits	Evidence	References
Consumer	Economic benefits	In the U.K., 13% of general practitioners and 5% of E.D. visits have been found to be reduced U.S. healthcare system saved approximately $146 billion/year from drug cost savings and clinical visit cost savings Cough/Cold/Flu, Upper GI, and Allergy segments saved approximately 59%	(4,10)
	Broadening consumer access	Nicotine replacement therapy (NRT) practice has increased by about 60% following reclassification, and a higher percentage of gum users had quit as compared to the pre-OTC period (9.7% vs. 14.6%, p = 0.05) Reclassification of Orlistat 60 mg (weight loss management drug) resulted in significant and satisfying weight loss without medical supervision	(11–13)
	Increase awareness	With OTC medications flow of information and disease awareness tends to increase, which is beneficial for overall disease management in a community	(14)
Regulators	Reduce healthcare burden	Decreasing health expenditure associated with the treatment of minor health conditions Improving the quality of care for serious diseases & focus on R&D, HCP’s can focus on burning issues due to reduction inpatient load	(10, 14)
	Progressive consumer health services	Mobile applications-based technologies providing health-based information about OTC medicines and spread awareness among consumers about their wellness	(15)
	Integral role of pharmacist	Selection of best OTC medication and monitoring of therapeutic outcome using diagnostic tests Reducing healthcare costs by preventing medication-related problems and errors Reducing the risk of possible adverse drug events and improving patient outcomes	(16)
Pharmaceutical companies	Lifecycle management	Extending the product lifecycle in market as compared to generic competitors	(17)
	Increase total sales revenues	Increase in sales revenues after Rx-to-OTC switches e.g., sales of Pepcid AC^®^ was more than $200 million in the first year after reclassification	(18)

### Regulatory process of prescription-to-OTC Switch

**Fig. 2:**
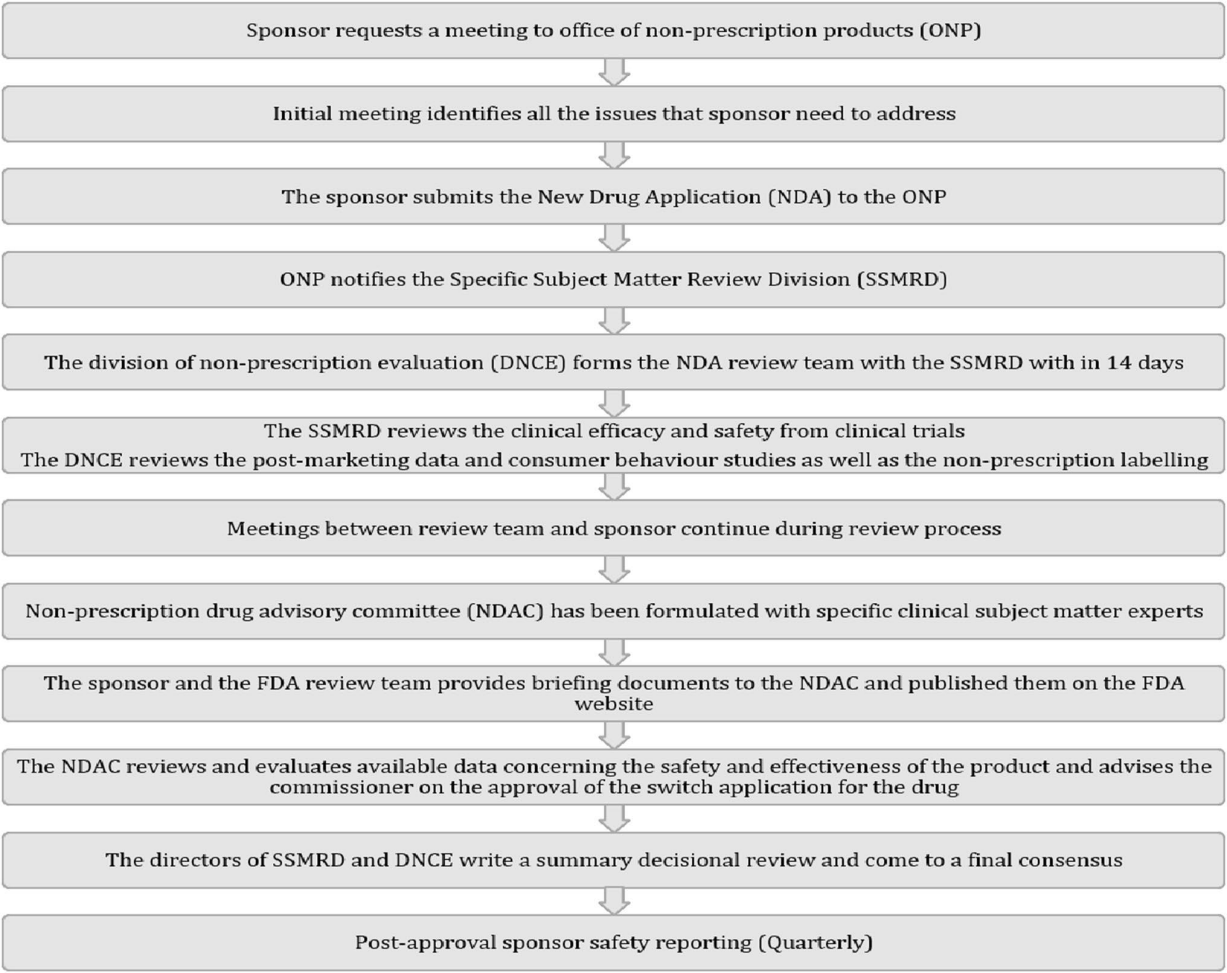
The typical review and approval Rx-to-OTC switch process in accordance with FDA guidelines

Generally, “Rx-to-OTC switch” is a strictly regulated, data-driven, and scientifically validated process. Well-documented guidelines and specific regulations have been developed globally to encourage and streamline the process of Rx-to-OTC switch. The most prominent countries implementing these guidelines are the United States, United Kingdom, New Zealand, Australia, Canada, and China. To apply for an Rx-to-OTC switch in the United States, a company files a New Drug Application (NDA) or an Abbreviated New Drug Application (ANDA) with the US FDA. The typical review and approval process for switches is detailed in Fig. 2 [17, 19]. It can take 12 months to several years to gain FDA approval for an Rx-to-OTC switch. Some switches require detailed consumer and self-selection studies to evaluate label comprehension. This data is required in addition to the standard efficacy and safety clinical trials that further validate the drug safety and efficacy in an OTC setting. Evidence-based reports in the form of post-marketing surveillance for new conditions of nonprescription use is an important data that need to be shared with advisory committees. Pharmacovigilance has played a pivotal role in the post-market analysis of new drugs. The legislative fundamentals of pharmacovigilance in India are directed by specifications mentioned in Schedule Y of the Drugs and Cosmetics Act 1945. The studies involve in post approval safety reporting includes Phase-IV studies and spontaneous report submitted by health professional and consumers. Furthermore, some switches are more straightforward to carry out, for example, if the drug is used to treat a symptomatic, acute, relatively benign condition. Conversely, drugs used to treat asymptomatic, severe, and chronic conditions are inherently more challenging to gain regulatory approval for the switch [20].

A defined OTC framework has been a long-standing industry ask in India, with pharma underscoring that it can improve access to treatments and support responsible self-medication to achieve multiple health and economic objectives. However, to date, India does not have a well-documented process or specific regulations with respect to switching Rx to OTC products. Indian regulator authority is likely to define and prepare a list of OTC drugs in due course. A well-defined OTC drug category has been outlined based on the recommendations given by Ahooja Committee and further approved by Drugs Consultative Committee (DCC). Draft rules recently put out by the country’s Ministry of Health and Family Welfare (MoHFW) has, for the first time, identified 16 drugs, which include antifungals, antihistamines, laxatives, and nasal decongestants, for inclusion in the OTC drugs category [21]. This new regulation could form the base for a switch policy for potential OTC drugs down the line [6].

## Redefining Indian healthcare perspective- a call for action

### Growing demand for OTC products and consumer trend in holistic healthcare solutions: Global & Indian insight

Growing consumer demand for OTC products can be witnessed through an inevitable surge in OTC drugs sale across the globe, especially during the pandemic that further exhibits the significant extent of their utilization. As a result, in 2021, the global OTC market size was worth USD 157.0 billion and is supposed to grow at a compound annual growth rate (CAGR) of 5.8% to reach USD 233.6 billion by 2028 [22]. Nevertheless, this global OTC drug market (5.8%) is low compared to the Rx drug market, which is assumed to grow at a CAGR of 8.9% from 2021 to 2028 [23].

**Fig. 3:**
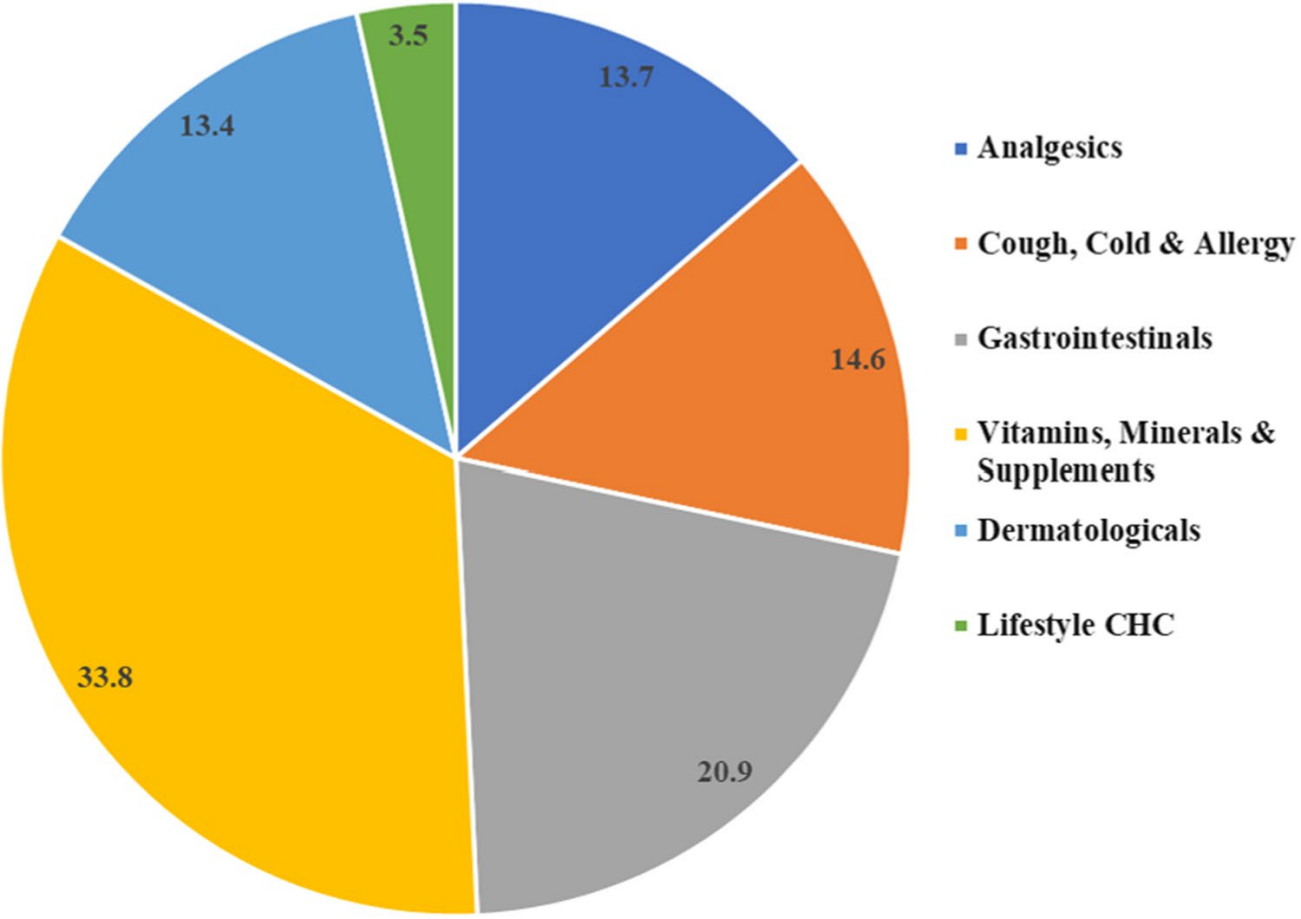
Market segments of various OTC products (Market research data available on Nicholas Hall’s Global CHC Database, DB6; 2021)

The Asia Pacific is inferred to showcase the highest CAGR during the forecast period (2021–2028), referring to OTC drug sales. In India, the OTC market is expanding due to a rise in healthcare expenditures, a growing population, and more healthcare awareness. It is dominated by products such as cold and cough, flu, dermatological, respiratory, analgesics, gastrointestinal, and VMS (vitamins, minerals, and supplements). The percent market share of various segments (2021) presented by Nicholas Hall’s Global CHC Database, DB6 showed market segments of vitamins, minerals, and supplements is 33.8%, followed by gastrointestinal having 20.9%, then cough, cold and allergy, analgesics and dermatological having 14.6%, 13.7%, and 13.5% respectively (Fig. 3). Likewise, herbal and Ayurveda-based products are also available as OTC medication and contribute to the overall growth rate of the OTC products category.

In 2020, the Indian OTC market size was worth USD 3.7 billion and has grown at a CAGR of 6.6% and attained a market size worth USD 3.9 billion in 2021 based upon market research data available on Nicholas Hall’s Global CHC Database, DB6) [24]. The Indian wellness-based products market has also been steadily growing over the past few years [25]. Most of the big pharmaceuticals players in India have apprehended the need of the Indian wellness market, and these pharmaceuticals companies are approaching the consumer need with a more significant portfolio of OTC products that can be marketed outside pharmacies, which eventually might lead to the development of more wellness-based OTC products. Holistic healthcare solutions are now considered as an integral part of mind and body wellness. The recent COVID-19 situation was the primary driving force behind this shift in attitude and cognizance about self-health care amongst consumers. In a study conducted by Tomar et al., 67.9% (n = 275) subjects out of 405 respondents revealed their solicitude and awareness regarding self-health care after the pandemic. Moreover, 79.6% (n = 19) have also started consuming various immunity booster products [26].

### Major driving force behind raising OTC practice

The trend of self-medication using OTC products is uprising in India [27]. Patient awareness towards self-medication, cost-effectiveness, easy accessibility, and product advancement are key factors contributing to the ever-increasing demand for OTC products [28].

India’s regulatory framework allows the advertisement of a few OTC products that assist in increasing disease awareness & subsequently broadening the access of OTC products to the larger population. Although facilities to purchase OTC drugs from non-pharmacies such as supermarkets and convenience stores, and gas stations are not available in India, unlike some countries, its pharma companies have now started to target post offices to sell their OTC product [29, 30]. This will lead to faster, broader, and economical access to these products to the rural population and wider community.

Product advancements that can meet consumer needs, also increase the demand for OTCs drugs [31]. Regulated E-cigarettes, nicotine patches, and Metho plus pain balm in a 5 g pack are a few examples. Also, pharmaceutical companies devote a significant amount of time and resources to developing new therapeutic areas and products in the OTC market in line with consumer necessity. For instance, a pharmaceutical company has introduced new formats for targeted applications (pain relief roll-on, etc.) [32]. Similarly, GSK consumer healthcare has introduced a new product called Otrivin Breathe Clean a Daily Nasal Wash in December 2020 and Iodex spray formulation for topical pain relief [33, 34]. Furthermore, Bengaluru-based consumer care company launched two OTC products for joint pain relief and smoking cessation in February 2019 [35].

Along with all these factors, HCP recommendations, use by a family member/family recommendation, country of origin, past experience with Rx from HCP, and the price of OTC medicine play an important role in consumer OTC preference and purchase behavior [36, 37]. This highlights the substantial role of HCPs or pharmacists towards mitigating the risk of potential adverse drug events as a significant influencer for selecting appropriate OTC medication for a specific indication, improving patient outcomes and healthcare savings across a variety of settings.

Ayurvedic drugs and traditional medicines, an integrative part of alternative system of medicine in India are sold over the counter freely by non-pharmacists. A steep rise into research and utilization of these medications for self-health care management was observed after COVID-19 pandemic. This trend has evolved notably by virtue of scientific evidence that supports its utilization to become an integral part of a country’s formal health care system. The shifting trend toward alternative system of medicine is also expected to propel the demand of OTC botanical medicine and to a limited extent towards sales of OTC phytomedicine [38, 39]. 

## Concerns/gaps with Self-medications utilizing OTC medications 

The risk associated with self-medication utilizing OTC medications, such as drug abuse, overdosing, misdiagnosis, drug interactions, polypharmacy, etc., impose several challenges and severe consequences on the healthcare system. In addition, it might bring about paradoxical economic loss of patients because of deceptive diagnoses and delay in appropriate treatment [40].

### Lack of awareness about OTC drugs

Although OTC drugs have proven efficacy and safety, their improper use due to lack of knowledge of the correct dose, side effects, and interactions could have profound implications, especially in children, pregnant women, and elders. Long-term administration of NSAIDs leads to several chronic diseases, such as acute renal failure, peptic ulcer disease, and stroke/myocardial infarction [41]. Also, concomitant administration of NSAIDs with some antihypertensives, antidepressants, and other commonly used medications may lead to severe adverse drug reactions (ADRs) because of drug-drug interactions (DDIs) [42]. The lack of stringent pharmaceutical regulatory standards in India has played a significant role in readily accessing and oversupplying various medications, including drugs with scant evidence of their safety profile data.

A study from Bangalore assessing the knowledge about adverse effects of drugs reported awareness in only 13% of the patients. Nearly 77% were not aware of contraindications or undesirable effects of the drugs. The authors also reported that nearly 85% of patients considered the information gained from the pharmacists to be sufficient. The disconcerting observation was during dispensing the OTC drugs, the pharmacists did not ask about the existing co-morbid conditions and drug allergy. Moreover, appropriate instructions were not given to all patients, and the adverse effects of the drugs were not explained. Drugs were not dispensed according to the appropriate dosage regimen. It can thus be concluded from the study that more awareness of patients and pharmacists about OTC drugs is required to prevent the harmful effects of the same [43].

### Labelling: gaps in adherence and awareness levels

The other important safety aspect of OTC drugs is the labeling requirement. The label or patient package insert provided with the drug act as a primary source of information for the patients for safe and effective drug administration. The basic information, which includes the drug’s name, use, dosage, and directions, should be part of the primary label. The U.S. Food and Drug Administration (FDA) has formulated stringent regulations to ensure the information provided on all OTC products. The label should contain all the required information in the same order as mentioned in the regulation document. In India, the label of a drug should confirm the specifications as per Drug and cosmetic Rule 97 of Drug and Cosmetic Act, 1945. However, mostly the instructions mentioned in labels are inadequate, incomplete, and missed by patients. It has been observed that approximately 90% and 96% of OTC labels provided inadequate & incomplete information about adverse drug effects and special instruction regarding its use in pregnancy and breastfeeding, respectively. The study also noted insufficient information in OTC medicine labels for the patient to make a “responsible” decision for self-medication. Sometimes, it doesn’t have the therapeutic category details and the dose that must be taken [44].

### Advertisement on product awareness/ adoption/ misuse

Advertisements have always been considered a novel way to spread the appropriate disease awareness and prudent sharing of drug-related information to consumers [45–47]. However, a deceptive advertisement might spread inadequate information and lead to irrational use. Thus, a closer examination of OTC drug advertisements by regulatory authorities seems essential, focusing on conveying quality and the essential information to consumers [48]. In India, the promotion of medicinal products is regulated by Drug and Magic (Objectionable Advertisement) Act, 1954 (DMRA).

### OTC distribution and access: India is still struggling to provide essential health care to remote places

Although India is home to approximately 1/6th of the world’s population and is expected to become world's most populous nation by 2050, the country is still struggling to provide essential health care to remote places. It has already been stated that around 67% of the Indian population currently lives in rural areas. Still, rural markets contribute only 17% of the sales, suggestive of limited access to OTC medicines. This could be a huge opportunity for improving access in rural settings that could enhance the reach of healthcare to all and act as the future growth drivers because rural markets are still unexplored markets for OTC medicines [49].

## Approaches to address the gaps: consumer centric innovations for driving access to timely self-care?

The increasing concerns/gaps of self-medication, such as drug abuse, overdosing, misdiagnosis, drug interactions, polypharmacy, etc., can be bridged using some of the strategies (used by other countries) as discussed below.

### Proper labelling of OTC medications

**Fig. 4:**
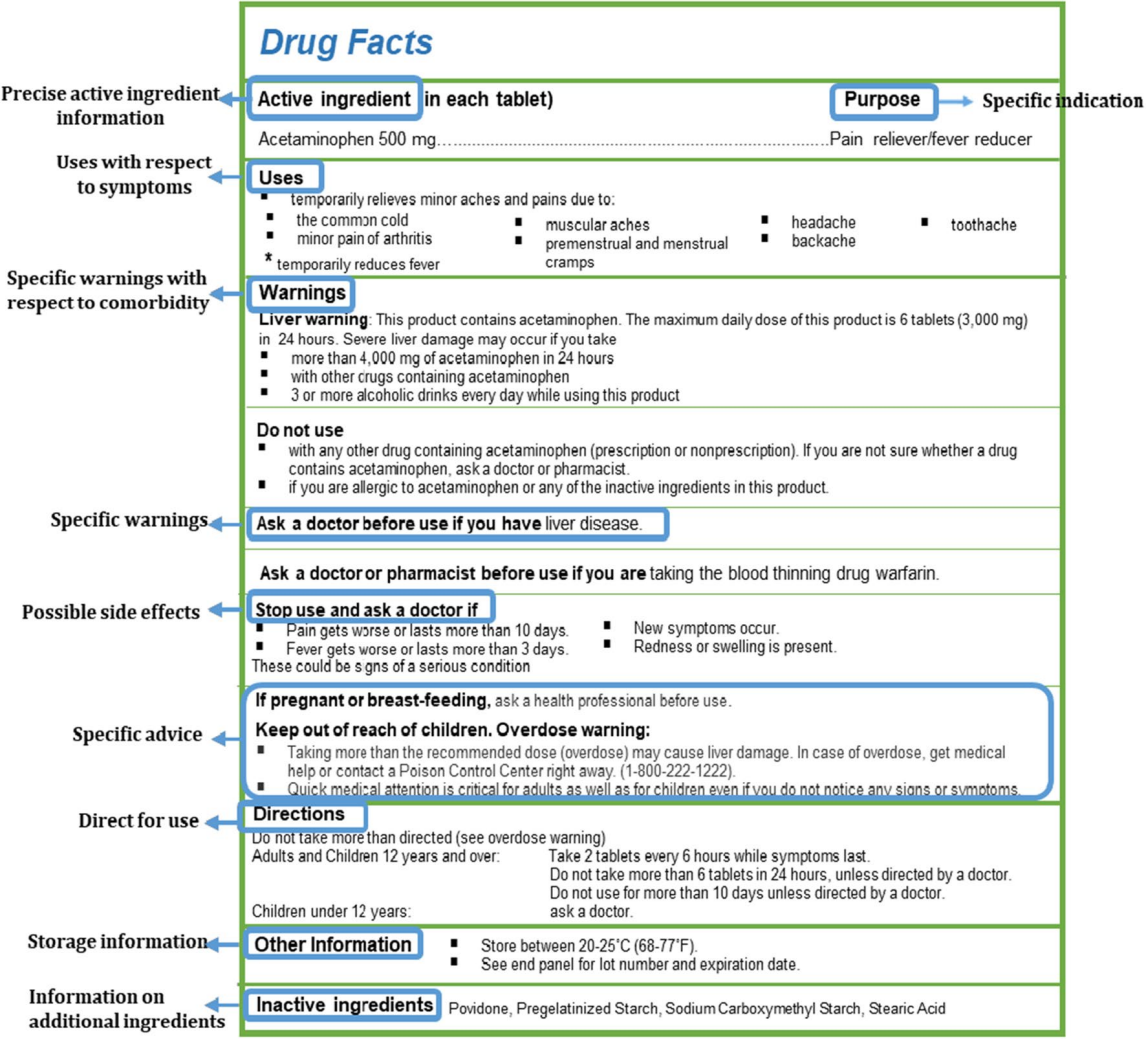
The Drug Facts label for the over-the-counter drug Acetaminophen includes information about ingredients, uses, warnings, and directions

The foremost challenge is the incomplete information on OTC pharmaceutical labels which ultimately leads to various impediments such as drug interactions, side effects, etc. In 2008, FDA passed a law stating that OTC medicines must have a new label called “Drug Facts.” It should include information on active ingredients, purposes, uses, warnings, directions for use, and inactive ingredients. This ensures that the patient uses the OTC medicine correctly and helps avoid potential adverse effects. Figure 4 depicts the Drug Facts label for the over-the-counter drug Acetaminophen includes information about ingredients, uses, warnings and directions.

In India, the required information on OTC pharmaceutical labels is also frequently insufficient for the patient to make an accountable self-medication decision, as it does not even include the therapeutic category and the dose that must be taken [44]. Thus, the approach of a new label called “Drug Facts” can be reciprocated in India for adequate and complete information on the label OTC medicine. Considering the diversity in languages spoken throughout India, apart from English, it will be helpful to provide label in local languages for OTC drugs. Furthermore, tamper-proof packaging can be introduced, ensuring easy identification of any tampering done with the OTC drug package label. Finally, adding a pictorial description on the label may be helpful to patients, especially those less educated.

### Raising awareness

During COVID-19, the general population began self-medication practices to safeguard against exposure to the virus. Under this scenario, it is necessary to raise awareness in public regarding irrational drug use [50]. In addition, pharmacist, and consumer awareness camps regarding the need for OTC or prescription medicine, side effects, dosage specification, and avoidance of polypharmacy may further streamline OTC management.

#### Consumer awareness for self-management

The major key pillars of a healthy OTC ecosystem are empowering consumers to make better, informed, and responsible choices. With a steep rise in mobile phone users in India, information technology and mobile application-based healthcare programs are a new development in the healthcare sector. A prevalent example of such a service is mHealth. mHealth is typically classified as having access to health-related queries and information using mobile phones and delivery of healthcare services [51]. A mHealth-based application (Epocrates / Epocrates Plus) is commonly used in the United States for drug-related information, safety profiles, generics, OTC medicines, and doses [52]. Similarly, Indonesian consumers use mHealth to search for trusted medical information, find the best hospitals, and deliver OTC medicine to their own houses [53]. In India, such applications are more prevalent in urban areas instead in rural extents. This is attributed to the challenges such as less awareness regarding smartphones, extensive power cuts, illiteracy rate, and reluctance towards advancement. A community-based strategy will be needed for awareness of the rural population. An effective consumer health ecosystem, including novel strategies, awareness camps, and OTC access, can possibly metamorphose the healthcare outlook in India. Such an approach also assists in upgrading healthcare access for the substantial unprivileged rural population and improving patient care for urban consumers. In the current scenario, consumers equipped with novel products and a more comprehensive understanding of healthcare solutions could yield a better and tranquil healthcare infrastructure.

#### Extended role of pharmacist in OTC medications

Pharmacists play an essential role in health care services, specifically in rural areas where physicians are not readily available. The community pharmacy service area is the only lifeline of the healthcare system or where primary care physician services are excessively high-priced. Moreover, Rx-to-OTC switches generate more possibilities for a pharmacist to serve the community by offering primary health care services to consumers.

Considering the increased accessibility of OTC medicines, role of pharmacists to monitor the potentially inappropriate use of OTC medicines has been increased. The pharmacist’s role is not only limited to monitor or suggest a safe and appropriate option tailored to customers’ need but may also advise an option related to non-pharmacological approaches, where medication seems to be avoidable. Moreover, pharmacist’s role to promote affordability is also vital by recommending a generic version as per consumer need.

Thus to strengthen pharmacy practices, Babar (2021) has presented an array of recommendations that can foster pharmacy practice in low and middle-income countries (LMICs) [54]. These recommendations are:

- Compulsory presence of graduate-level pharmacists at community pharmacies
- Clear demarcation of the functions and obligations of a graduate and diploma pharmacists
- Effective categorization and execution of medicines into (a) prescription drugs (b) pharmacists only drugs (c) over the counter medicines
- Implementations of stringent regulation controlling the sale of medicines
- Forbidding doctors from dispensing medicines
- Implementation of Universal Health Coverage Schemes that involve pharmacies and pharmacists and improve the affordability of medicines
- Strengthen the role of national medicines regulatory authorities
- Educate pharmacists with required clinical skills and minor ailment schemes
- Enforcement of national medicines information strategy to provide appropriate medicine information to healthcare professionals and consumers
- Mandatory Continuing Professional Development (CPD) programs for the Pharmacists.

### Need to develop the new ways to distribute and deliver the OTC products

New ways to distribute and deliver OTC drugs to the rural population must be considered. India has almost 1,50,000 post offices and 9,00,000 pharmacy shops. If the services of post offices are used, the reach will dramatically increase. However, if the drugs are provided in places or platforms other than the pharmacy shop, then conditions necessary for proper storage must be strictly followed. The online distribution of OTC drugs and products can create a new niche of drug distribution, which will further enhance the access to essential medicine to a broader reach. In a study conducted in 2017, 61% of German internet users purchased through online pharmacies, having 73% of these medicines being in the OTC category [55]. As a significant player in retail e-commerce, Amazon achieved the highest sales, with a rise of 28% in 2019 with OTC healthcare and beauty products. A similar approach has been followed by Lazada and Ali Baba [55]. This conviction of lower cost and well-informed consumer demand propelled e-commerce and improved access to OTC products.

### Introduction of robust OTC drug policy

The utilization of OTC medicines in India could be further streamlined through a robust policy that can be attained with the help of well-defined regulations, pharmacist support, patient awareness programs, and the active role of pharmaceutical companies. A few recent articles and media reports highlighted that the Indian government is now giving utmost consideration to OTC regulations, and the term “OTC” has been recommended as a distinct category of drugs [6].

## OTC medication regulation -India and global scenario

### Global scenario- a lesson to learn

Developed countries such as the United Kingdom, Australia, Germany, and Japan have devised specific regulatory guidelines considering the non-obvious differentiation between OTC and prescription medicines. The different categories of drugs according to their classifications are discussed in Table 2 [56].

**Table 2:** Drug classification categories among countries^56^

Country	Regulations	Categories of drug	Subcategories
U.S	The United States Federal Food, Drug, and Cosmetic Act	Prescription drug	Prescription drug
		Over the counter drug	Over the counter drug^#^
UK	Medicine Act 1968	Prescription drug	Prescription only drug
		Over the counter drug	Pharmacy Drugs**
			General sale list drugs^#^
Singapore	Health Product Act	Prescription drug	Prescription only drug
		Over the counter drug	Pharmacy Drugs**
			General sale list drugs^#^
Malaysia	Poison Act 1952	Prescription drug	Prescription (Group B)
		Over the counter drug	Group C**
			Non-scheduled poisons^#^
Canada	National Drug Schedule Program	Prescription drug	Prescription (Schedule 1)
		Over the counter drug	Schedule 1
			Schedule 2**
			Schedule 3*
			Unscheduled^#^
Japan	Pharmaceutical Affairs Act	Prescription drug	Pharmacy Drugs
		Over the counter drug	Guidance mandatory drug^##^
			Type-1 non-prescription drugs^##^
			Type-2 non-prescription drugs^#^
			Type-3 non-prescription drugs^#^
Philippines	Philippine Pharmacy Act	Prescription drug	Dangerous drug Rx**
			Exempted dangerous drug Rx
			Prescription drug
		Over the counter drug	Over the counter drugs**
			Household remedies^#^
Australia	Therapeutic Goods Act 1989	Prescription drug	Prescription only drug
		Over the counter drug	Pharmacy medications (Schedule 2)^#^
			Pharmacist only medications (Schedule 3)**
			General sales medications^#^
Thailand	The Drug Act (No.6) A.D. 2019	Prescription drugs	Controlled drug
		Over the counter drug	Dangerous drugs**
			Non-dangerous drugs*
			Household remedies^#^

^#^General sale medicine or sales can be done by a non-pharmacist employee; *Open-shelf medicines; **behind the counter drugs; ^##^transition drug category and require pharmacist dispensing to evaluate the safety of drug

In addition, the classification and uses of OTC have been considered carefully. This concept can be further explained with the help of ibuprofen sales in the U.S. and New Zealand. The low-dose ibuprofen viz. 200 mg is treated as an OTC product that helps to manage minor illnesses such as headaches. However, high-dose ibuprofen viz. 400–800 mg, used to treat severe illnesses, mostly arthritis pain, is considered a prescription medicine.

Table 3 represents examples of a few common drugs in eight countries with different classifications [56]. The table shows the examples of drugs that had a wide range of drug classifications, such as prescription, behind-the-counter (BTC), Open-shelf medicines (OPS), and general sale medicine (GSL) drug categories.

**Table 3:** Drug Classification of few drugs having different status across different countries^56^

Indication	Drug	Dose	US	UK	JP	SG	MY	PH	CA	TH
Hypertension	HCTZ	50 mg tab	P	P	P	P	P	P	P	BTC/OPS^1^
Fungal infection	Clotrimazole	1% cream	P/GSL^2^	BTC/GSL^2^	P/T^2^	GSL	GSL	P	GSL	OPS
	Ketoconazole	2% cream	P	P/BTC/GSL^2^	P	BTC/GSL^2^	BTC	GSL	P	BTC
	Ketoconazole	200 mg tab	P	P	–	P	P	–	P	BTC
Pain	Ibuprofen	200 mg cap	GSL	BTC/GSL^2^	P/GSL^2^	P/BTC/GSL^2,3^	BTC	BTC	GSL	BTC
	Ibuprofen	400 mg tab	P	P/BTC/GSL^2^	–	P/BTC	BTC	BTC	GSL	BTC
Allergy	Cetirizine	10 mg tab	GSL	P/BTC/GSL^2^	P/GSL^2^	P/GSL^2^	BTC	BTC	GSL	BTC
	Cetirizine	1 mg/mL syrup	P/GSL^2^	BTC/GSL^2^	–	BTC/GSL^2^	BTC	BTC	OPS	BTC
	Loratadine	10 mg tab	GSL	P/BTC/GSL^2^	P/T^2,4^	BTC/GSL^2^	BTC	BTC	GSL	BTC/OPS^5^
	Desloratadine	5 mg tab	P	P	P	P/BTC	BTC	P	GSL	BTC

^1^This drug is classified as OPS if it is contained in four or ten tablet-packaging with a designated warning. Otherwise, it is classified as BTC. ^2^Each brand of these drugs was classified into different categories. ^3^These drugs are granted exemptions for supply without a prescription as BTC drugs if certain criteria are met. ^4^This drug was reclassified from “prescription” to “transitional drugs” on 17 January 2017. ^5^This drug is classified as BTC unless it meets certain criteria, which are in divided solid dosage forms for oral use containing 10 mg or less per dose with the label “only for a season allergic rhinitis, not for runny nose from the common cold” when sold in the manufacturer’s original packaging containing not more than 10 tablets per strip, 2 strips per carton

*US* the United States, *UK* the United Kingdom, *JP* Japan, *SG* Singapore, *MY* Malaysia, *PH* the Philippines, *CA* Canada, *TH* Thailand, *P* prescription drugs, *BTC* behind-the-counter drugs, *T* transition drugs, *OPS* open-shelf drugs, *GSL* general sale list drugs, *tab* tablets, *cap* capsules, *loz* lozenges

### Regulatory landscape evolving in India

#### OTC drugs: current regulatory perspectives and need for policy

Self-care and self-medication utilizing OTC drugs has the potential to do good as well as cause harm since it involves the use of drugs. The benefits associated with OTC medication include faster and cheaper access, less burden on the health care system, and consumer involvement. However, adverse drug reactions and drug abuse are some of the contradictory aspects. Thus, there should be a mechanism in place to ensure that proper safety precautions will not be compromised by means of promoting self-medication.

Many countries recognize OTC medicines as a separate category of drugs and have established regulations for their use. In India, till date, there are no guidelines for licensing OTC medicines. There is no separate category allotted for OTC medicines in India, and the drugs that do not come under the prescription medicines schedule are generally sold as over-the-counter medicines. A few household remedies such as paracetamol, eucalyptus oil, liquid paraffin, tincture, and some cough and cold formulation covered under Schedule K of the Drug and Cosmetic Act and its rules are potential OTC drugs. In addition, some medicines covered under Schedule K of the Drug and Cosmetics can be dispensed by non-chemists or non-drug licensed stores where the population is below 1000 subjects.

Similarly, topical or external administration of a few Schedule G and H drugs is not covered under the prescription category. For example, diclofenac is a schedule H drug when administered orally. On the other hand, topical diclofenac is not a schedule H drug. Likewise, considering the side effects and fatality in dengue patients using aspirin, the State Government of Delhi in 2017 announced reassigning aspirin as a prescription drug from the “Household Remedies” list. There is also well-formulated regulation under the Drugs and Magic Remedies (objectionable advertisement) act to avoid self-medication of some drugs/classes [57].

#### Updates in OTC drug regulations: government and regulatory committee’s steps

In 2017, the Drugs Consultative Committee (DCC) suggested the conception of specific rules and regulations to govern the categorization and directive of OTC drugs. This led to the creation of a subcommittee (Ahooja committee) to comprehensively examine the drugs covered under various Schedules, i.e., Schedules H, H1, G, X, and K, in the Drugs & Cosmetics Act, 1940 (D&C Act) and the Drugs and Cosmetics Rules, 1945 (D&C Rules). Moreover, the committee needs to identify the list of drugs that can be sold under the OTC category in India [28]. Later in 2019, DCC recognized that the subcommittee examined the matter in detail and submitted the report. The subcommittee undermined the various aspects such as the definition of OTC drug, essential characteristics and classification of OTC drugs, list of drugs identified and related regulations, regulation of Rx to OTC switch process, and regulations of new OTC drug approval and manufacturing, labelling, distribution, advertisement, pricing and sale of OTC drugs. Finally, the meeting held on August 20, 2019, the DCC highlighted the vital need for categorizing OTC drugs and formulating robust provisions for regulation of OTC drugs. The list of recommendations given by the Ahooja committee is below:

- Promote self-care without compromising patient safety, thereby reducing treatment costs.
- Lay down the definition for OTC drugs in the D&C Rules.
- Incorporate basic characteristics of OTC drugs.
- Classify OTC drugs into OTC-1 and OTC-2 based on the extent of evidence of safety, therapeutic index, need for accessibility to patients, availability, non-habit-forming nature, present supply-chain mechanism, and socio-economic conditions of the country.
- Prepare an initial list of OTC drugs.
- Regulate the switch of prescription drugs to OTC drugs.
- Regulate new OTC drug approval.
- Regulate the distribution and sale of OTC drugs.
- Regulate the advertisement of OTC drugs.

In accordance with the recommendations given by Ahooja Committee, the DCC advised for the necessary amendments in Schedule K of the D&C Rules to include necessary appropriations for OTC drugs to provide an exemption from requirements of prescription and/or sale license, depending upon appropriate conditions. The DCC also suggested Ahooja Committee to identify the list of OTC drugs, together with conditions, and frame the draft for amending the D&C Rules. A recent update has highlighted that the government has proposed introducing an OTC list with 16 medicines but has not described the exclusion or inclusion criteria [58].

While it appears that the DCC firmly believes that OTC drugs should be regulated like a separate category of drugs, regulatory changes in either the D&C Act or the D&C Rules had not been approved yet.

In order to boost the OTC market, OPPI's committee is now working to encourage responsible self-medication. It also directed to raise awareness about the significance of appropriate self-medication through community education and awareness campaigns. The committee not only encourages the use of over-the-counter medications but also emphasizes their safety. In addition to supporting OTCs, a balance must be struck between broader and narrower interests [59].

## Conclusion

Self-care and self-medication utilizing over-the-counter drugs have become crucial components of healthcare, but their availability can act as a double-edged sword, for instance, inadvertent drug usage during the COVID-19 epidemic. Inappropriate self-medication and the use of OTC drugs might result in an inaccurate self-diagnosis, major untoward side effects, drug interactions, drug dependence, and resistance to pathogens. Considering the safety of the consumers, the Indian government is now giving utmost importance to OTC regulations, and the term “OTC” has been recommended as a distinct category of drugs. Accurate labeling of products and pharmacists education will help consumer awareness and their safety, and the introduction of a robust OTC drug policy is the need of the hour to further ensure efficient access for selfcare and improved healthcare outcomes. All stakeholders need to come together and join hands in this endeavor to optimize usage, adoption and access to OTC medicines in India.

## Data Availability

Not applicable.

## Abbreviations

OTC: Over-the-counter
HCPs: Healthcare Professionals
WHO: World Health Organization
NDA: New drug application
FDA: Food and Drug Administration
ANDA: Abbreviated New Drug Application
DCC: Drugs Consultative Committee
MoHFW: Ministry of Health and Family Welfare
CAGR: Compound annual growth rate
VMS: Vitamins, minerals, and supplements
NSAIDs: Nonsteroidal anti-inflammatory drug
ADR: Adverse drug reactions
DDIs: Drug-drug interactions
DMRA: Drug and Magic (Objectionable Advertisement) Act, 1954
LMICs: Low and middle-income countries
BTC: Behind-the-counter
GSL: General sale medicine
OPS: Open-shelf medicines
P: Prescription drugs
US: The United States
UK: The United Kingdom
JP: Japan
SG: Singapore
MY: Malaysia
PH: The Philippines
CA: Canada
TH: Thailand
